# Human cytomegalovirus epidemiology and relationship to tuberculosis and cardiovascular disease risk factors in a rural Ugandan cohort

**DOI:** 10.1371/journal.pone.0192086

**Published:** 2018-02-06

**Authors:** Lisa Stockdale, Stephen Nash, Angela Nalwoga, Hannah Painter, Gershim Asiki, Helen Fletcher, Robert Newton

**Affiliations:** 1 London School of Hygiene and Tropical Medicine, Faculty of Infectious and Tropical Diseases, London, United Kingdom; 2 London School of Hygiene and Tropical Medicine, Faculty of Epidemiology and Population Health, London, United Kingdom; 3 Medical Research Council/Uganda Virus Research Institute, Entebbe, Uganda; 4 University of York, Department of Health Sciences, York, United Kingdom; 5 International Agency for Research on Cancer, Lyon, France; University of San Francisco, UNITED STATES

## Abstract

Human cytomegalovirus (HCMV) infection has been associated with increased mortality, specifically cardiovascular disease (CVD), in high-income countries (HICs). There is a paucity of data in low- and middle-income countries (LMICs) where HCMV seropositivity is higher. Serum samples from 2,174 Ugandan individuals were investigated for HCMV antibodies and data linked to demographic information, co-infections and a variety of CVD measurements. HCMV seropositivity was 83% by one year of age, increasing to 95% by five years. Female sex, HIV positivity and active pulmonary tuberculosis (TB) were associated with an increase in HCMV IgG levels in adjusted analyses. There was no evidence of any associations with risk factors for CVD after adjusting for age and sex. HCMV infection is ubiquitous in this rural Ugandan cohort from a young age. The association between TB disease and high HCMV IgG levels merits further research. Known CVD risk factors do not appear to be associated with higher HCMV antibody levels in this Ugandan cohort.

## Introduction

Human Cytomegalovirus (HCMV), also known as human herpesvirus-5 (HHV-5), is a member of the β-herpes virus family which is widely distributed in human populations. HCMV transmission occurs through person-to-person contact. It can be transmitted transplacentally to neonates or through breast milk of an infected and shedding mother, by intimate contact and by transplantation from (or sharing syringes with) an infected individual [1]. It has been shown that young children shed HCMV virus in saliva and urine at high levels which may add to transmission between infants and adult caregivers [2].

Congenital HCMV infection is the leading cause of permanent hearing and neurological impairment as well as vision loss in infants worldwide [3]. Maternal primary infection or reactivation, especially during the first trimester, is particularly associated with adverse neonatal outcomes [4]. The incidence of congenital HCMV infection is estimated at between 0.7 and 5% of all births in low- and middle-income countries (LMICs) [5].

In immunocompetent adults, HCMV infection rarely causes disease; however, once infected, the virus remains latent in a wide range of cell types, including lymphocytes and myeloid lineage cells, as well as smooth muscle cells and endothelial cells which line blood vessels [6]. HCMV/HIV co-infection is common and is an important cause of HCMV retinitis and severe non-AIDS events, including death, in HIV-infected individuals [7,8]. HCMV infection is associated with chronic immune activation [9] and recent evidence implicates immune activation with increased risk of tuberculosis (TB) disease [10].

Epidemiological studies in high-income countries (HICs) have found associations between HCMV infection and increased risk of mortality in older people [11,12]. Further studies have implicated chronic HCMV infection as a risk factor for cardiovascular disease (CVD); a recent meta-analysis of studies conducted in HICs, estimated a 22% increased relative risk of CVD with exposure to HCMV [13]. In a UK setting, HCMV infection was associated with the development of arteriosclerosis [14] and a 3mmHg increase in systolic blood pressure among older individuals [15]. As Africa undergoes what has been described as an ‘epidemiological shift’ from infectious to non-communicable disease [16], an estimated 1.2 million deaths in Africa were attributed to CVD in 2004 [17].

Associations of HCMV and CVD have not been fully investigated in LMICs where HCMV infection rates are much higher than in HICs [18–20]. Studies from other herpes viruses associated with non-communicable disease (NCD) in areas where infection is ubiquitous (such as EBV in relation to African Burkitt lymphoma and KSHV in relation to African Kaposi Sarcoma), show that risk of NCD increases with increasing viral antibody titre [21–24].

In this study, we investigate HCMV seroprevalence in a large cross-sectional rural Ugandan cohort (n = 2,174) and investigate associations with co-infections, clinical measurements and demographic information. As a secondary analysis, we link cardiovascular risk factors to HCMV antibody levels.

## Material and methods

### Study area and design

The General Population Cohort (GPC) is a population-based open cohort study, set up in 1990 by the Medical Research Council (MRC) UK in collaboration with the Uganda Virus Research Institute (UVRI). Initially to examine trends in HIV prevalence and incidence, the GPC is located in Kyamulibwa sub-county of Kalungu district, rural south-western Uganda [25]. The cohort now comprises a cluster of 25 neighboring villages with approximately 20,000 residents (52% aged ≥13 years) from three ethnic groups, the majority (75%) being from the Baganda tribe, the main tribal group in the region.

Blood samples are transported to MRC/UVRI laboratories in Entebbe where a portion of the venous blood sample is analysed according to protocol guidelines. Remaining samples are stored at –80°C in a biobank in Entebbe [25].

The majority of samples tested for HCMV in this study were taken from 2011. Samples from TB cases were from a range of GPC rounds sampled between 1999 and 2014. Active pulmonary TB was diagnosed through positive sputum smear microscopy after passive case detection.

### Sampling

Individuals were selected at random, having been stratified by age and sex. Infants under 5 years of age were oversampled in anticipation of high age-dependent HCMV seropositivity based on evidence from other sub-Saharan countries. A target sample size of approximately 100 individuals per year of age from under 1 year to 5 year were sampled and 200 individuals in 5 to 10 year age groups thereafter. The sex ratio was approximately equal within each age group (Table 1).

**Table 1:** General population cohort characteristics of individuals included in this study.

Age, mean (range)	22.7 (0.08–100.75)
Sex, percentage female (number/total)	50.2% (1,091/2,174)
HIV prevalence, percentage (number/total)	4.6% (100/2,134)
Body mass index*, percentage (number/total)	
- Underweight	21% (237/1,150)
- Normal	69% (797/1,150)
- Overweight	8% (97/1,150)
- Obese	2% (19/1,150)
Hypertension**, percentage (number/total)	
- Normal	46% (552/1,187)
- Pre-hypertension	39% (460/1,187)
- Hypertension	15% (175/1,187)
Tribe, percentage (number/total)	
- Baganda	75% (1,524/2,035)
- Rwandese Ugandan	16% (319/2,035)
- Other §	9% (192/2,035)

*body mass index 18.4 and below is underweight, 18.5–24.9 is normal, 25.0–29.9 is overweight, and 30 and above is obese.

** Pre-hypertension is systolic blood pressure (SBP) between 120mmHg and 140 mmHg, and a diastolic blood pressure (DBP) between 80 mmHg and 90 mmHg. Hypertension is defined as SBP ≥140mmHg and DBP ≥90mmHg.

§ Other tribes include Bakiga, Batooro, Banyankole, Basoga, Bafumbira, Tanzanian, Barundi

The age structure of sampling for this study was determined based on Ugandan demographics; 49% of Ugandans are under 14 years of age, 21% are between 15–24 years of age, 28% between 25 and 64 years of age and only 2% over 65. The sampling here was similar to the Ugandan demographic with 45% under 14 years, 18% 15–24, 32% 25–64 and 5% over 65 years of age.

Being a cross-sectional study, each individual was only sampled once. Siblings and parent-child pairs were not excluded.

### Ethics

Written consent for the use of clinical records and biological samples for research purposes was obtained from all GPC participants following Uganda National Council of Science and Technology guidelines.

Ethical approval for the use of samples for this study was obtained from The UVRI Research and Ethics Committee and from the Uganda Council for Science and Technology, in addition to the London School of Hygiene & Tropical Medicine, London, UK.

### HCMV serology

Serum samples were tested for antibodies against HCMV using standard, validated and commercially available enzyme-linked immunosorbent assay (ELISA) against HCMV IgG (IBL International GmbH). Testing was conducted at UVRI in Entebbe. Briefly, serum samples were thawed and diluted as per kit instructions to 1:100 in sample diluent. One hundred microliters of diluted serum and controls in duplicate were dispensed into a 96-well plate coated with HCMV antigen. Plates were covered and incubated for 1hr at 37°C and then washed before 100μL of anti-IgG conjugate was added and the plate covered and incubated again for 30 minutes at room temperature. After another washing step, 100μL of tetra-methyl-benzidine (TMB) substrate solution was added and the plate incubated for 15 minutes in the dark at room temperature before 100μL of 0.2M sulphuric acid solution was added to stop the reaction. Plates were read at 450/620nm within 30 minutes to obtain optical density (OD), a surrogate marker of antibody titer.

Individuals were considered to be seropositive if the IgG OD measurement of plate controls fell within the kit specifications and the mean of duplicate measurements was above the calculated cut off.

### Linking to GPC data

Testing for HIV was carried out immediately after blood collection in Uganda as previously described by Asiki *et al* [25]. Data were linked to the samples retrieved via each participant’s unique GPC identifier.

Anthropometric measurements and blood sampling were performed by trained interviewers/nurses using calibrated instruments following standard protocol guidelines. Body mass index (BMI) was calculated as weight (kg) divided by height (m)^2^. Pre-hypertension was defined as having a systolic blood pressure (SBP) between 120mmHg and 140 mmHg, and a diastolic blood pressure (DBP) between 80 mmHg and 90 mmHg. Hypertension was defined as having a SBP ≥140mmHg and DBP ≥90mmHg. Proportion hypertensive was calculated by dividing the number of hypertensive individuals by the combined number of normal and pre-hypertensive individuals. Classification of BMI into four categories and definition of hypertension were derived from current World Health Organization (WHO) and National Institute of Health guidelines, respectively [26,27]. Biochemical analyses were performed using the Cobas Integra 400 plus analyser to determine lipid profiles for total cholesterol, high and low density lipoproteins (HDL and LDL respectively) from serum samples, and HbA1c from whole blood samples. Abnormal lipids were defined as raised total cholesterol being > 5.2mmol/L, low HDL as < 1.0mmol/L for men, and < 1.3mmol/L for women [28].

Total IgG was measured by ELISA utilizing a commercial standard curve. Degraded samples with negative total IgG were excluded from the study.

Samples used in this study were linked to demographic and other clinical data corresponding to the same time period at which the sample was taken.

### Statistical analysis

Participants were categorized as either HCMV seronegative or seropositive based upon OD results; seropositive samples were further categorized into tertiles of OD, to differentiate low, medium and high responders. The Student’s t test was used to compare continuous variables (such as HCMV IgG antibody OD measurement) between two independent groups (sex, tribe (Baganda or other), HIV and TB status). Associations of HCMV IgG OD with age, tribe, sex, HIV and TB were investigated. An analysis of variance (ANOVA) was used to compare continuous variable outcome between HCMV tertiles.

Linear regression was used to determine the relationship between HCMV IgG OD (as the dependent variable) and sex, HIV and TB status (as the independent variables–tribe was excluded in HCMV IgG OD regression analysis due to missing data– 10/27 active TB cases did not have information on tribe), and also between CVD risk factors with a continuous measurement (dependent variables: HbA1c, cholesterol, HDL, LDL and BMI) and the independent variables HCMV tertile, age and sex. Logistic regression was used for the binary outcome variables HCMV seropositive/seronegative and proportion hypertensive. Due to the approximate two-fold increase in relative risk of coronary heart disease or acute myocardial infarction in HIV-infected patients [29–31], associations between HCMV and CVD variables are limited to HIV negative individuals in this analysis.

HCMV OD data were slightly positively skewed, possibly resulting in artificially narrower confidence intervals (CI) around estimates. Cardiovascular disease (CVD) risk measurements were available for individuals over the age of 12 years.

To account for evidence of non-linearity of HCMV OD with age (likelihood ratio test p = 0.0025), a quadratic term for age was included in the regression analyses.

To account for multiple comparisons, 99% CI are reported and a p value of 0.01 is considered to represent strong evidence to reject the null hypothesis.

Data were entered using Microsoft Excel and analysed using STATA version 14 (Stata Corporation, College Station, TX, USA).

## Results

Of the 2,189 samples tested, 15 had negative total IgG levels and so were excluded due to probable degradation of sample, resulting in 2,174 samples being included in initial analyses. Following these exclusions, 40 individuals did not have a valid HIV result and so were excluded from regression analysis where HIV was included as a regression term.

Overall 91% (1,988/2,174) of the population tested positive for HCMV antibodies. Among children less than 15 years of age, 1% (10/991) were HIV positive; among individuals over 15 years of age, 8% (90/1,143) were HIV positive. The highest proportion infected with HIV (15%) was within the 41–50 year age group.

### Factors affecting HCMV IgG antibody levels

**Fig 1:**
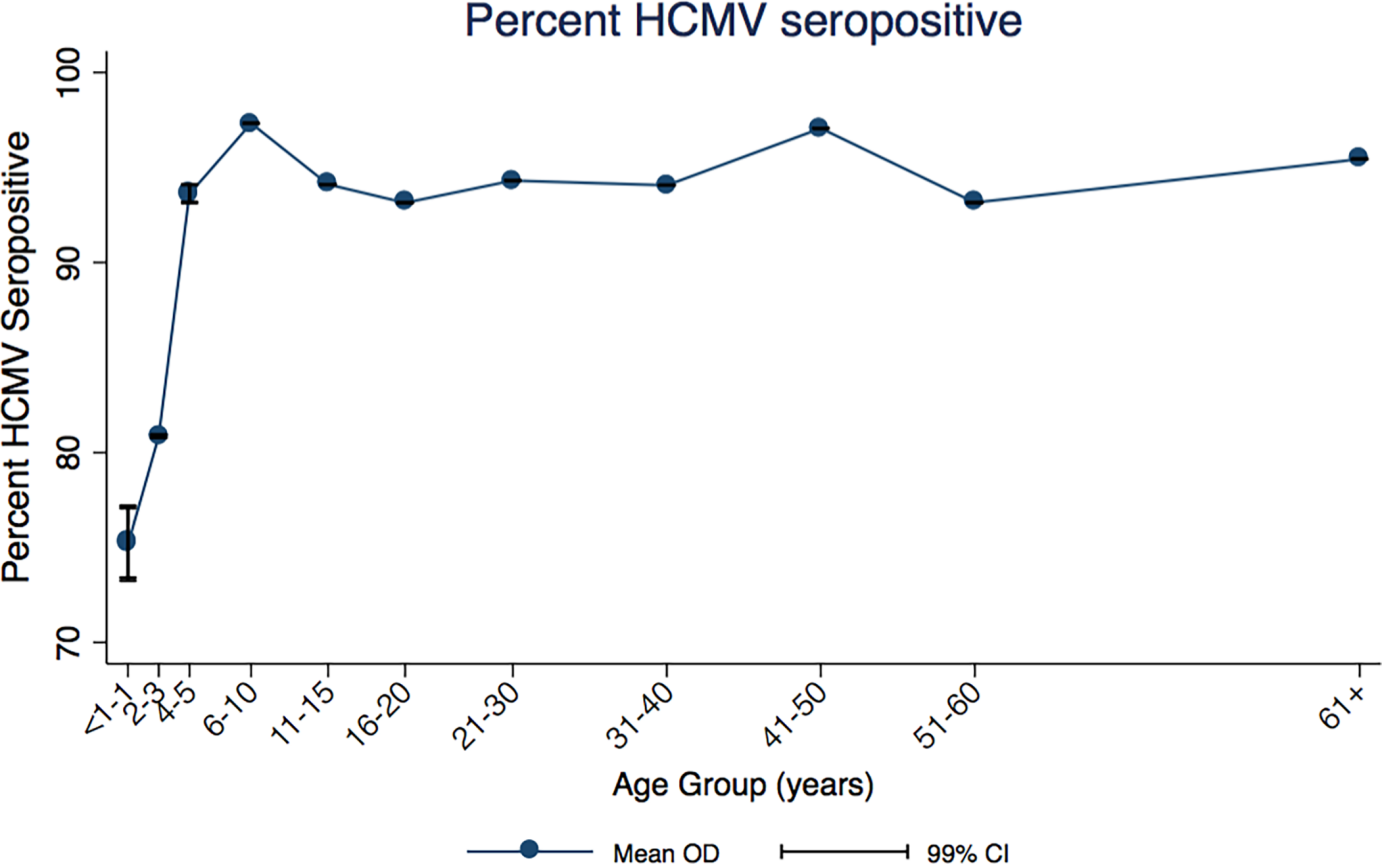
Percent HCMV seropositive by age group (years). Vertical lines show 99% CI for each mean data point. CI—confidence interval, HCMV—human cytomegalovirus.

The percentage of HCMV seropositive individuals in this population increases for both males and females equally from birth until the 6–10 year age group, after which seropositivity levels plateau at around 95% (Fig 1): 83% HCMV seropositivity was seen by age one year and this increased to 95% by age five.

Because of the ubiquity of HCMV infection within this population it was decided to conduct further analyses on the HCMV seropositive population only. An analysis of the HCMV negative population (n = 186, none of whom were TB cases and only 4 HIV positive) showed no significant associations for sex or HIV in regression analysis. The only significant association was with age in years (p<0.001).

**Fig 2:**
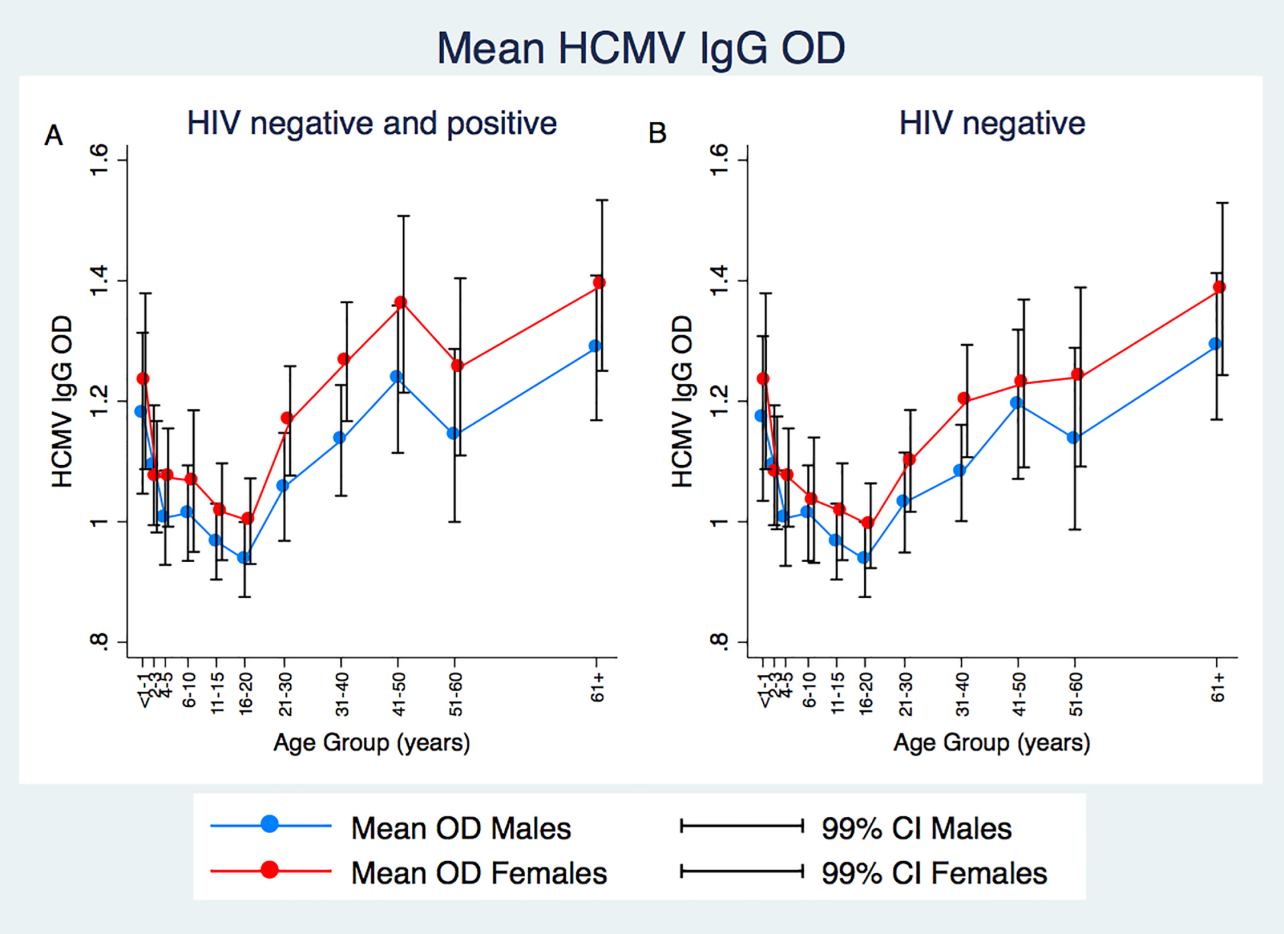
**Fig 2A. Mean HCMV IgG OD levels in HIV negative, HCMV seropositive individuals by age group (years) and sex. Fig 2B. Mean HCMV IgG OD levels in HIV negative and HIV positive individuals combined by age group (years) and sex.** Red lines show female mean values, blue shows male mean values. Vertical lines show 99% CI for each mean data point. CI—confidence interval, HCMV—human cytomegalovirus, HIV—human immunodeficiency virus.

Of the HCMV seropositive individuals, IgG levels initially decrease from a peak in those <1 year until the 16–20 year age group, after which HCMV IgG levels increase for both males and females (Fig 2A). When HIV negative individuals are plotted separately, the increased HCMV IgG levels in the 31–50 year old groups coincide with the highest HIV positivity (Fig 2B).

To investigate impact of age on HCMV IgG levels, ages were further split into < 9 months, 9 months to 12 years, 13–20 years, 20–60 and 60 years and above. Age groups selected are based on presence of maternal antibodies (conservative estimate of 9months [32]), prior to sexual debut (9 months—12 years [33]), post sexual debut (13–20 years [33]), 21–59 years and post 60 years. It was found that infants under the age of 9 months have higher mean HCMV IgG (1.25 OD) than any other age group apart from the over 60 years olds (1.34 OD) in unadjusted analyses.

A 0.07 OD increase in HCMV IgG (99% CI 0.02,0.12, p<0.001) is associated with being female in unadjusted analyses. This increase remained after adjusting for age, HIV and TB infection status (mean difference 0.07 OD 99% CI 0.03,0.11, p<0.001) (Table 2). The 0.49 OD increase in HCMV IgG associated with HIV infection also remained after adjustment for age, sex and TB infection status (mean difference 0.47 OD 99% CI 0.37,0.57, p<0.001) (Table 2).

**Table 2:** Unadjusted and fully adjusted mean differences (values obtained using a multivariable model including age, quadratic age, sex, HIV and TB status) in HCMV IgG OD with p value (t test for unadjusted values, regression for adjusted values) and 99% confidence intervals.

Factor (n)	HCMV IgG					
	Unadjusted mean difference	P value	99% CI	Adjusted mean difference	P value	99% CI
Sex						
Male (978)	baseline			baseline		
Female (978)	0.07	<0.001	0.02, 0.12	0.07 §	<0.001	0.03, 0.11
HIV †						
Negative (1,860)	baseline			baseline		
Positive (96)	0.49	<0.001	0.40, 0.60	0.47	<0.001	0.37, 0.57
TB ‡						
Negative (1,929)	baseline			baseline		
Positive (27)	0.34	<0.001	0.15, 0.53	0.19	0.006	0.01, 0.37

CI—confidence interval, HCMV—human cytomegalovirus

†HIV—human immunodeficiency virus

‡TB–Active pulmonary Tuberculosis.

**Fig 3:**
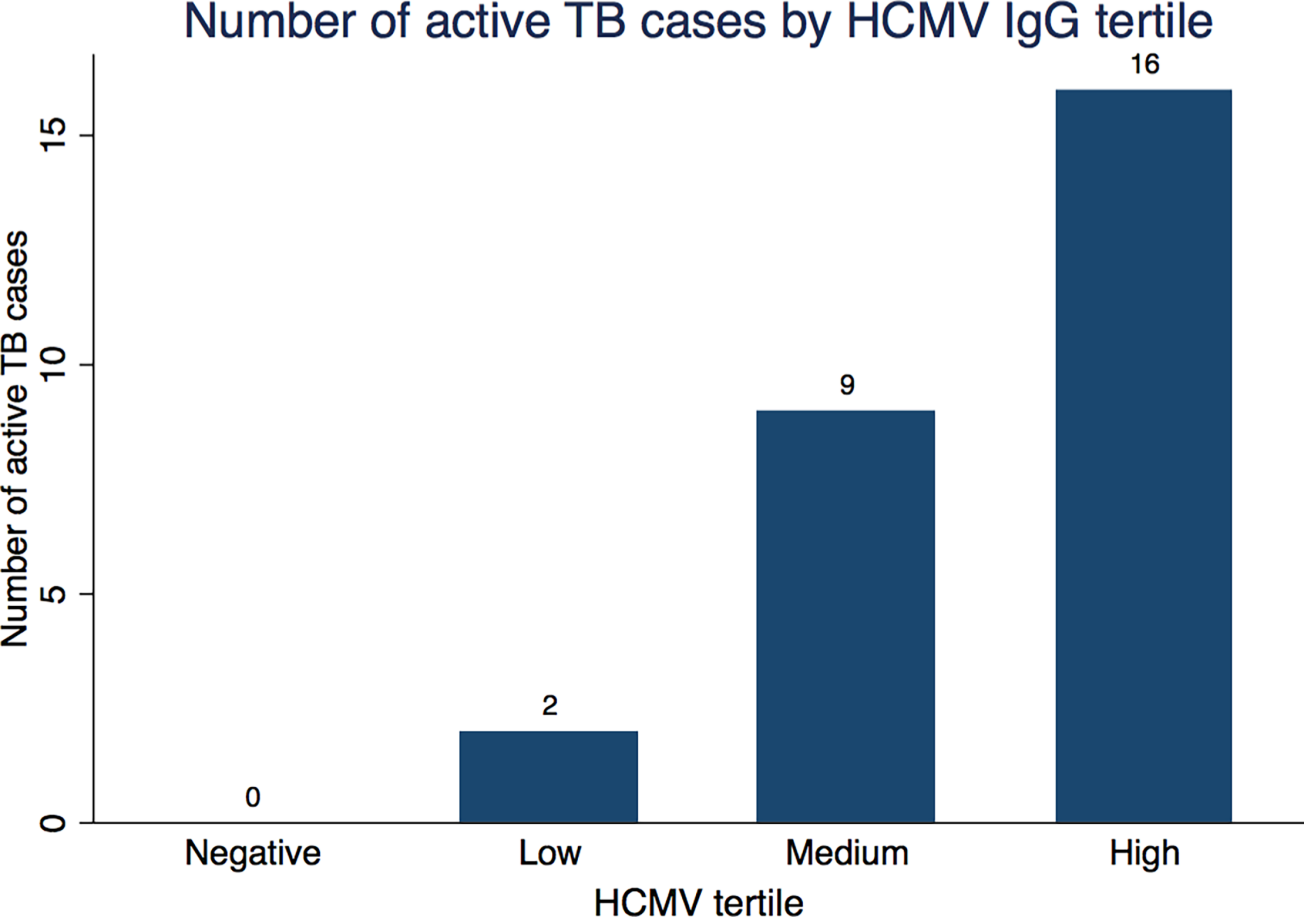
Number of active TB cases by HCMV IgG tertile. Numbers above bars show number of active TB cases (n = 27 total active TB cases).

Mean HCMV IgG OD levels among the 27 people with sputum-confirmed pulmonary TB was 0.34 OD higher than individuals without active TB. After adjusting for age, sex and HIV infection status this difference is partly explained by those variables but there remains good evidence of that active TB disease is associated with an increased HCMV level of 0.19 OD (99% CI 0.01,0.37, p = 0.006) (Table 2) which equates to a 7% increase based the range of HCMV OD values (min 0.31, max 2.84 OD). When HCMV seropositivity was divided into low, medium and high responders, the majority of active TB cases had high HCMV IgG levels (16/27, 59%). Thirty three percent were of medium HCMV IgG (9/27), 7% (2/27) had low HCMV, and none were HCMV seronegative (Fig 3).

When HIV positive and negative individuals are analysed separately, the effect of active TB disease is shown to be greater in the HIV positive group, but the direction of effect is the same. Among HIV negative people (S1 Table), having active TB disease is associated with an adjusted increase of 0.11 OD of HCMV IgG (99% CI -0.10–0.33, p = 0.170) whereas among the HIV positive individuals (S2 Table), having active TB disease is associated with an increased HCMV IgG OD of 0.32 OD (99% CI -0.12,0.77, p = 0.063).

Being from a tribe other than the majority Baganda tribe was associated with a 0.14 OD increase in HCMV IgG (99% CI 0.09,0.19, p<0.001). Due to missing data (10/27 active TB cases did not have information on tribe), and the fact that inclusion of tribe into regression analyses did not alter mean difference coefficients, this variable was not included in regression analyses. Although not included, a sensitivity analysis resulted in a change in coefficient for sex from 0.068 to 0.070 OD, a change in coefficient for hiv from 0.466 to 0.454 and a change in coefficient for TB from 0.193 to 0.184. The coefficient for tribe was 0.11 OD 99% CI 0.06–0.16.

### Association of HCMV IgG levels with cardiovascular disease risk factors

Cardiovascular risk factors were assessed in individuals over 12 years of age (mean age of individuals included in CVD risk factors analysis: 36.5 years, range 12–100 years) due to availability of data.

Because of the known increased risk of CVD in HIV-infected individuals, associations between HCMV and CVD predictors is limited to HIV negative individuals in this analysis, who represented 94% (2,034/2,174) of the total sample.

Of the 2,034 HIV negative individuals included in this analysis, data for cholesterol, HDL, LDL, and mean SBP and DBP were available for 58% (1,186) of individuals, HbA1c levels for 58% (1,176) and BMI measurements were available for 57% (1,150) of individuals, all of whom were over the age of 12 years.

Among the individuals for whom BMI data was available in this cohort, 21% (237/1,150) were underweight, 69% (797) normal weight, 8% (97) overweight and 2% (19) obese. Of the 1,0186 individuals with measured blood pressure, 15% (175) were hypertensive, 39% (460) were pre-hypertensive, and 46% (552) were not hypertensive (Table 1).

**Fig 4:**
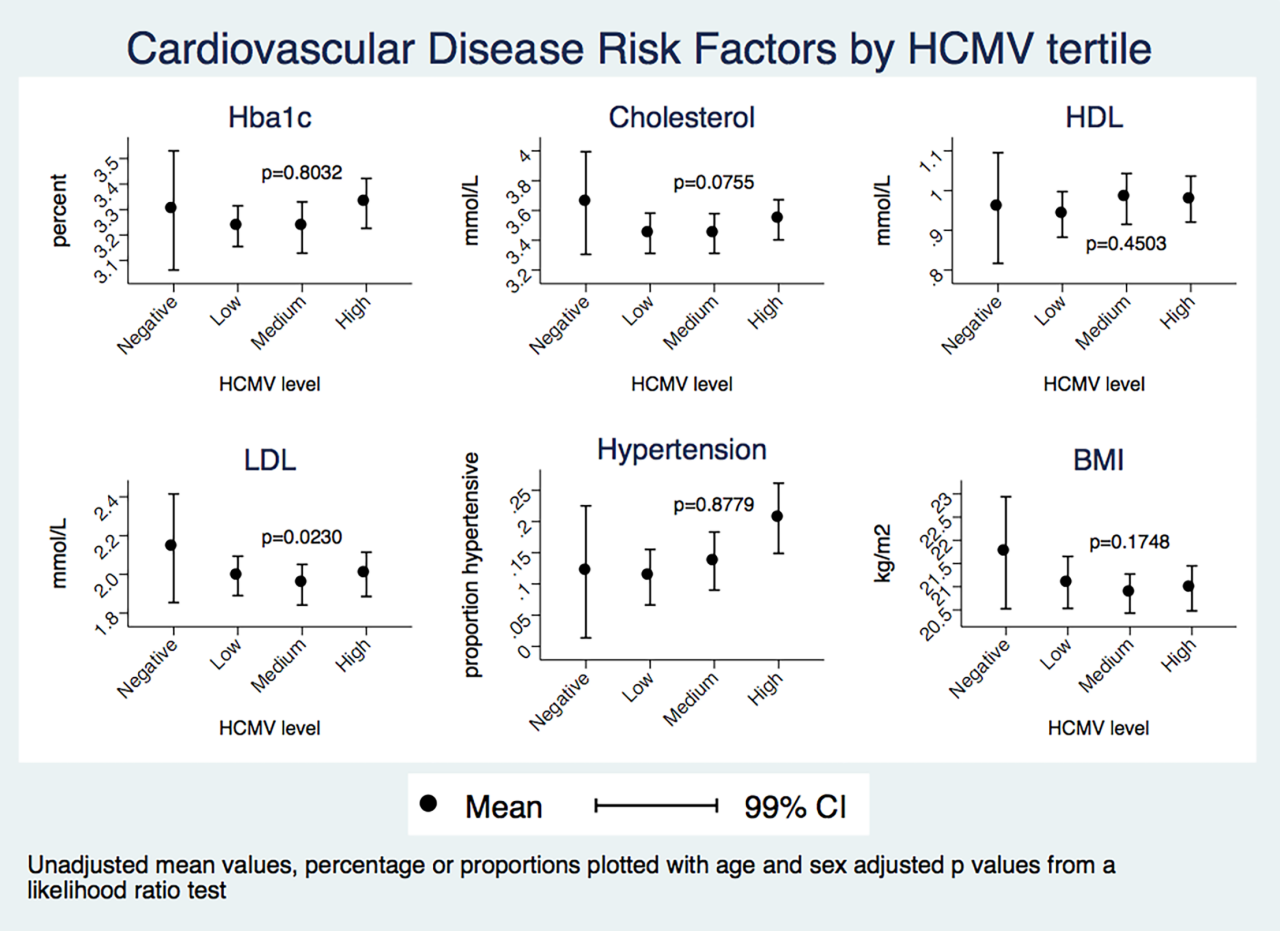
Bar graphs showing unadjusted mean CVD risk factors by HCMV IgG OD level. **HIV positive individuals were excluded from this analysis. Mean and 99% CI with p value from linear regression or logistic regression (for hypertension). p values shown are from age- and sex-adjusted likelihood ratio tests.** CI—confidence interval, CVD—cardiovascular disease, HCMV—human cytomegalovirus, HIV—human immunodeficiency virus.

Initial regression analyses showed no evidence of a difference between CVD risk factors between HCMV seropositive and HCMV seronegative individuals in a model adjusting for age and sex. When HCMV seropositive individuals were further divided into low, medium and high responders, the only CVD risk factor that showed an association with HCMV IgG levels in univariate analysis was hypertension, which is more prevalent in people with medium or high HCMV IgG levels (Fig 4). After adjusting for age and sex however, this association is explained by these variables (Table 3). Addition of tribe as an explanatory variable in regression analyses did not alter coefficients.

**Table 3:** Age and sex-adjusted regression for CVD risk factors cholesterol, HbA1c, HDL, LDL and BMI and hypertension showing mean difference (or Odds Ratio), p value from likelihood ratio test and 99% CI.

	HbA1c (%)			Cholesterol (mmol/L)			HDL (mmol/L)			LDL (mmol/L)			Hypertension (proportion)			BMI (mg/kg2)		
HCMV level	Mean difference	p value	99% CI	Mean difference	p value	99% CI	Mean difference	p value	99% CI	Mean difference	p value	99% CI	Odds ratio	p value	99% CI	Mean difference	p value	99% CI
Negative	baseline			baseline			baseline			baseline			baseline			baseline		
Low	-0.032	0.803	-0.26, 0.20	-0.113	0.076	-0.43, 0.20	0.007	0.450	-0.14, 0.15	-0.080	0.023	-0.34, 0.18	1.073	0.878	0.34, 3.35	-0.485	0.175	-1.69, 0.72
Medium	-0.072		-0.30, 0.16	-0.172		-0.49, 0.14	0.025		-0.12, 0.17	-0.159		-0.41, 0.10	1.095		0.36, 3.34	-0.736		-1.93, 0.46
High	-0.046		-0.28, 0.19	-0.266		-0.58, 0.05	-0.028		-0.17, 0.12	-0.234		-0.49, 0.02	1.256		0.42, 3.78	-0.910		-2.12, 0.30

CI—confidence interval, CVD—cardiovascular disease, HDL—high density lipoproteins, LDL—low density lipoproteins, BMI—body mass index

## Discussion

HCMV is an important pathogen in congenital neurological conditions and in the context of immune suppression. Recent studies have suggested a link between HCMV seropositivity and excess overall mortality, specifically from CVD, within HIC settings [12–15,34]. Relatively little attention has been paid to determining HCMV prevalence and associations with non-communicable disease in LMICs.

Here, we find that 95% of this rural Ugandan population are seropositive for HCMV by age five years (Fig 1). This finding corroborates other findings in sub-Saharan African settings where high levels of HCMV seropositivity have been found; 96% in Egypt [35], 97% in Benin [36], 99% in the Gambia [37] and 86% in South Africa [38].

Among the HCMV seropositive population in this study we see high levels of HCMV IgG antibody in young infants under the age of nine months (Fig 2). While this may be a true reflection of congenital or primary infection of infants, IgG is the only antibody isotype that can be transferred through the placenta [39], and so these high levels may be driven by the inability of the assay to distinguish between maternal and ‘self’ IgG. Detection of virus as opposed to antibodies would allow elucidation during the first nine months of life while maternal antibodies persist.

After initial high levels of HCMV IgG in infants under the age of one year, we see a rapid decline whereby maternal antibodies are lost, or primary infection is controlled (but individuals remain seropositive), followed by a gradual increase from the age of 16 years of age up to the highest OD levels seen after 40 years of age.

The magnitude of antibody response to virus after maternal antibodies have waned is indicative of intensity of exposure. The finding that females have higher HCMV antibody levels compared to males after adjusting for age, HIV and TB (Table 2) was surprising. There is evidence that risk factors throughout life result in a higher risk of HCMV transmission in women compared to men; a study conducted in Uganda using data on breast feeding trends, showed that being a male baby increased the risk of early termination of breastfeeding compared to females [40]. Findings from another Ugandan longitudinal cohort found that age at sexual debut has been consistently lower for women than for men from the 1950’s to 1990’s [33]. In addition, the infant caregiving role of females results in disproportionate female interaction with infants during the peak shedding at 1–2 years of age [41].

Our results suggest that HIV infection and active TB disease are independently associated with increased levels of HCMV IgG antibodies (Table 2). HIV infection is correlated with hypergammaglobulinaemia by way of defective humoral immunity resulting in hyperactivated naive B cells producing large quantities of IgG [42]. Here we see an association between HIV infection and elevated IgG specific to HCMV, however in the same cohort, we see increased total IgG as well as tetanus toxoid-specific IgG in HIV-infected individuals, an association which remained after adjusting for age and sex (Stockdale *et al*, manuscript in preparation). Despite this potential confounding of HIV infection upon IgG levels, the increased levels of HCMV IgG in HIV infected individuals is corroborated by epidemiological evidence pointing towards high levels of HIV and HCMV co-infection due to similar routes of transmission [7].

Both TB and HIV are known to increase general inflammation [43,44], an immune environment which is associated with the increased likelihood of HCMV reactivation [45]. Both reactivation and reinfection expose the immune system to HCMV antigens, thereby resulting in elevated levels of HCMV-specific IgG antibodies.

The association of active pulmonary TB with raised HCMV IgG levels was in contrast to findings of a hospital-based study where HCMV-specific T-cell responses in a whole blood assay were found to be lower in TB patients who died when compared to TB patients who survived [46]. In this hospital-based study, HCMV responses were likely a general indictor of ‘immune fitness’ and therefore predictive of patient survival. By contrast, our study was community based and the severity of TB disease at the time of blood draw would have been low when compared to a hospital-based study.

The association of active pulmonary TB with raised HCMV IgG in our study was consistent with the findings of a correlates of TB risk study in South African infants where risk of active TB disease was increased with an activated T-cell phenotype which was itself correlated with T-cell interferon gamma production upon stimulation with HCMV antigens [10]. Neither active case finding, nor routine latent TB infection screening is conducted in the Ugandan GPC and therefore we cannot be certain that ‘non-TB’ individuals here have not been exposed to TB or indeed that they are not latently infected with *Mycobacterium tuberculosis*. Despite there only being 27 active TB cases within this cohort, there remains strong evidence of an association between TB and elevated HCMV IgG after adjusting for age and sex; an association which appears to be intensified by co-infection with HIV (S1 and S2 Tables).

In view of the 1.5 million deaths per year associated with TB [47], the association of increased HCMV IgG with active TB disease may be an important future research area, especially in areas with large numbers of people living with HIV.

It is important to note that the data from this study gives us no information on causality. Longitudinal studies measuring systemic viral loads along with antibody levels to HCMV (and other herpes viruses) in stored sera would be useful to understand progression of both herpes virus and TB disease in this cohort.

### Association of HCMV with CVD risk factors

Rates of CVD are increasing in sub-Saharan Africa [48]. In 2014, the WHO estimated that 27% of all deaths in Uganda were attributable to non-communicable diseases; specifically 9% of all deaths to CVD [49]. In HICs it has been postulated that the increased overall mortality associated with HCMV infections is associated with vascular morbidity [34]. HCMV infection has been implicated in increased arterial stiffness in chronic kidney patients [14] and in older individuals in the UK [15], and there is growing evidence of an important role for HCMV-induced inflammation in vessel walls [50] through persistent replication of HCMV in endothelial cells [6].

The current literature reports conflicting evidence for an association of HCMV infection with increased risk for CVD mortality. Of the ten studies included in a recent meta-analysis which found an excess risk of ischemic heart disease, stroke, and cardiovascular death with HCMV exposure [34], none were carried out in sub-Saharan Africa or other LMICs (studies were from United States (4 studies), United Kingdom (3 studies), Canada (1 study), Sweden (1 study), and Italy (1 study)). Another large meta-analysis comprising three studies and 9,657 patients found an overall significant association between HCMV positivity and hypertension (79% of the hypertension patients were HCMV positive compared with 64% of the controls–(Odds Ratio (OR) 1.39, 95% CI 0.95,2.05, p = 0.017) [13]. Of the three studies included, one was carried out in the USA, one in Iran and one in China. The overall OR masks large heterogeneity in the data, with the Iranian study showing high levels of HCMV seropositivity (93% overall seropositivity) and no association, while the US study reported 55% HCMV seropositivity and association between HCMV and hypertension among males only [13]. In one US population, association of HCMV seropositivity with all-cause mortality remained after adjusted analysis, however specific association with CVD mortality disappeared after adjusting for confounders [51] and in another, an association with hypertension was largely explained away by age [52].

The aforementioned studies investigated HCMV seropositivity in relation to CVD risk factors. In our study we investigate magnitude of antibody response to HCMV in a near-ubiquitously infected population. We do not see an association between the burden of HCMV infection, as measured by IgG, and CVD risk factors. It may be that the association between HCMV and CVD is masked due to burden of other chronic and acute infections in these communities. However more studies are needed to determine any association with excess mortality in populations with ubiquitous HCMV infection, and to assess if risk factors found to be important in one population are useful indicators of risk in other populations. Indeed, many studies have found reference standards developed in Europe and North America unhelpful when studying other populations [53,54]. It is essential that measurements are clinically relevant to the population in question, and that clinical research institutions ensure that standardized methods are used if data are to be comparable across countries and continents.

Despite the young age of individuals in this cohort (mean age 36.5 years, range 12–100 years), we do know that CVD outcomes (such as stroke) are not uncommon [55] and so these analyses could be expected to show association with HCMV antibodies if any exist. Evidence from a large cohort study in the US found that CVD risk factors linked to myocardial infarction and coronary heart disease death in later life are measurable in individuals as young as 20 years of age [56].

Despite an established link between HCMV serum antibody levels and blood viremia (and viral shedding in urine and saliva) among children [57], and the link between high systemic viral copy number and increased risk of mortality in HIV positive adults [58], it may be of interest in future studies to link viral load and risk factors to active HCMV disease in this population. Due to limited sample volume here, we were unable to measure viral DNA copy number in this study.

Considering the paucity of studies linking HCMV and all-cause mortality in LMIC settings and the association seen here between HCMV and TB, we believe it will be important to conduct longitudinal studies to elucidate these associations further in areas with almost universal prevalence of HCMV infection, specifically in sub-Saharan Africa.

### Limitations

The GPC has no official vital registration system and so we do not have CVD outcomes to link to CVD risk factors. Future studies which specifically investigate CVD related deaths within the GPC would be helpful to link HCMV antibody levels in longitudinal retrospective analyses using stored sera. The GPC is perfectly placed to collect CVD outcome data in future rounds of data collection and so these outcome measures can be linked back to clinical measurements and stored biological samples.

## Supporting information

S1 Table Unadjusted and fully adjusted mean differences (values obtained using a multivariable model including age, quadratic age, sex and TB status) in HCMV IgG OD with p value (t test for unadjusted values, regression for adjusted values) and 99% confidence intervals for HIV negative individuals only (n = 1,860). (DOCX)

S2 Table Unadjusted and fully adjusted mean differences (values obtained using a multivariable model including age, quadratic age, sex and TB status) in HCMV IgG OD with p value (t test for unadjusted values, regression for adjusted values) and 99% confidence intervals for HIV positive individuals only (n = 96). (DOCX)
