# Supplementary material for: Human cytomegalovirus epidemiology and relationship to tuberculosis and cardiovascular disease risk factors in a rural Ugandan cohort
Source: PLoS One. 2018 Feb 6;13(2):e0192086. doi: 10.1371/journal.pone.0192086 (PMC5800673; doi:10.1371/journal.pone.0192086)
Supplement: S1 Table — (DOCX) [file pone.0192086.s001.docx]

**S1 Table. Unadjusted and fully adjusted mean differences (values obtained using a multivariable model including age, quadratic age, sex and TB status) in HCMV IgG OD with p value (t test for unadjusted values, regression for adjusted values) and 99% confidence intervals for HIV negative individuals only (n=1,860).**

| Factor (n) | HCMV IgG | | |  |  |  |
| --- | --- | --- | --- | --- | --- | --- |
|  | Unadjusted mean difference | P value | 99% CI | Adjusted mean difference | P value | 99% CI |
|  |  |  |  |  |  |  |
| Sex |  |  |  |  |  |  |
| Male (941) | baseline |  |  | baseline |  |  |
| Female (919) | 0.06 | <0.001 | 0.01,0.10 | 0.06 | <0.001 | 0.02, 0.10 |
| TB ‡ |  |  |  |  |  |  |
| Negative (1,842) | baseline |  |  | baseline |  |  |
| Positive (18) | 0.13 | 0.142 | -0.09-0.35 | 0.11 | 0.170 | -0.10, 0.33 |

CI - confidence interval, HCMV - human cytomegalovirus, ‡TB – Active pulmonary Tuberculosis.
