# Supplementary material for: Human cytomegalovirus epidemiology and relationship to tuberculosis and cardiovascular disease risk factors in a rural Ugandan cohort
Source: PLoS One. 2018 Feb 6;13(2):e0192086. doi: 10.1371/journal.pone.0192086 (PMC5800673; doi:10.1371/journal.pone.0192086)
Supplement: S2 Table — (DOCX) [file pone.0192086.s002.docx]

**S2 Table. Unadjusted and fully adjusted mean differences (values obtained using a multivariable model including age, quadratic age, sex and TB status) in HCMV IgG OD with p value (t test for unadjusted values, regression for adjusted values) and 99% confidence intervals for HIV positive individuals only (n=96).**

| Factor (n) | HCMV IgG | | |  |  |  |
| --- | --- | --- | --- | --- | --- | --- |
|  | Unadjusted mean difference | P value | 99% CI | Adjusted mean difference | P value | 99% CI |
|  |  |  |  |  |  |  |
| Sex |  |  |  |  |  |  |
| Male (37) | baseline |  |  | baseline |  |  |
| Female (59) | 0.18 | 0.087 | -0.09, 0.45 | 0.14 | 0.174 | -0.13, 0.42 |
| TB ‡ |  |  |  |  |  |  |
| Negative (87) | baseline |  |  | baseline |  |  |
| Positive (9) | 0.38 | 0.030 | -0.07-0.83 | 0.32 | 0.063 | -0.12, 0.77 |

CI - confidence interval, HCMV - human cytomegalovirus, ‡TB – Active pulmonary Tuberculosis.
